# The Impact of Intermittent Hypoxemia on Left Atrial Remodeling in Patients with Obstructive Sleep Apnea Syndrome

**DOI:** 10.3390/life12020148

**Published:** 2022-01-20

**Authors:** Yung-Lung Chen, Yung-Che Chen, Hui-Ting Wang, Ya-Ting Chang, Yen-Nan Fang, Shukai Hsueh, Wen-Hao Liu, Pei-Ting Lin, Po-Yuan Hsu, Mao-Chang Su, Kuo-Tung Huang, Meng-Chih Lin

**Affiliations:** 1 Department of Internal Medicine, Division of Cardiology, Kaohsiung Chang Gung Memorial Hospital, Kaohsiung 833, Taiwan; feymanchen@gmail.com (Y.-L.C.); wideopen1216@ocm.tw (Y.-N.F.); pather@cgmh.org.tw (S.H.); wenhao@cgmh.org.tw (W.-H.L.); r40391132@gmail.com (P.-T.L.); 2 School of Medicine, College of Medicine, Chang Gung University, Taoyuan 333, Taiwan; yungchechen@yahoo.com.tw (Y.-C.C.); gardinea1983@gmail.com (H.-T.W.); emily0606@cgmh.org.tw (Y.-T.C.); 3 Graduate Institute of Clinical Medical Sciences, College of Medicine, Chang Gung University, Taoyuan 333, Taiwan; 4 Department of Internal Medicine, Division of Pulmonary & Critical Care Medicine, Kaohsiung Chang Gung Memorial Hospital, Kaohsiung 833, Taiwan; hsupowan@yahoo.com.tw (P.-Y.H.); maochangsu@yahoo.com.tw (M.-C.S.); jelly@cgmh.org.tw (K.-T.H.); 5 Emergency Department, Kaohsiung Chang Gung Memorial Hospital, Kaohsiung 833, Taiwan; 6 Department of Neurology, Kaohsiung Chang Gung Memorial Hospital, Kaohsiung 833, Taiwan

**Keywords:** intermittent hypoxemia, left atrial remodeling, obstructive sleep apnea syndrome, inflammation

## Abstract

Obstructive sleep apnea syndrome (OSAS) is a significant risk factor for left atrial (LA) remodeling. Intermittent hypoxemia occurs during the sleep cycle in patients with OSAS and plays a crucial role in cardiovascular pathologies such as stroke, arrhythmia, and coronary artery disease. However, there is very little information about the role of intermittent hypoxemia in LA remodeling in patients with OSAS. In total, 154 patients with sleep-related breathing disorders (SRBD) were prospectively recruited for this study. All enrolled SRBD patients underwent polysomnography and echocardiography. Significant OSAS was defined as an oxygen desaturation index (ODI) of ≥10 per hour. Intermittent hypoxia/reoxygenation (IHR) stimulation was used to test the effect of hypoxia on the viability, reactive oxygen species, apoptosis, and inflammation-associated cytokine expression in the HL-1 cell line. To investigate the effect of patients’ exosomes on HIF-1 and inflammation-associated cytokine expression, as well as the relationship between ODI and their expression, exosomes were purified from the plasma of 95 patients with SRBD and incubated in HL-1 cells. The LA size was larger in patients with significant OSAS than in those without. There was a significant association between ODI, lowest SpO_2_, mean SpO_2_, and LA size (all *p* < 0.05) but not between the apnea–hypopnea index and LA size. IHR condition caused increased LDH activity, reactive oxygen species (ROS) levels, and apoptosis in HL-1 cells and decreased cellular viability (all *p* < 0.05). The expression of HIF-1α, TNF-α, IL-6, and TGF-β increased in the IHR condition compared with the control (all *p* < 0.05). The expression of HIF-1α, IL-1β, and IL-6 increased in the HL-1 cells incubated with exosomes from those patients with significant OSAS than those without (all *p* < 0.05). There was a significantly positive correlation between ODI and the expression of HIF-1α, TNF-α, IL-1β, IL-6, and TGF-β; a significantly negative correlation between mean SpO_2_ and IL-6 and TGF-β; and a significantly negative correlation between the lowest SpO_2_ and HIF-1α (all *p* < 0.05). In conclusion, intermittent hypoxemia was strongly associated with LA remodeling, which might be through increased ROS levels, LDH activity, apoptosis, and the expression of HIF-1α and inflammation-associated cytokines.

## 1. Introduction

Left atrial (LA) remodeling is linked to cardiovascular disease, ischemic stroke, and mortality [1,2,3]. The most prevalent and easily observable clinical characteristic of structural remodeling is LA enlargement, which is triggered by LA pressure and volume overload [4]. Inflammation cytokines, inflammation-related structural alterations, and fibrosis have all been linked to LA remodeling [5]. Several inflammatory cytokines, including interleukin (IL)-1β, IL-6, tumor necrosis factor (TNF)-α, and transforming growth factor (TGF)-β, were discovered to be linked to atrial fibrosis and could be utilized to predict clinical outcomes [6,7,8,9,10].

The most common type of sleep-related breathing disorder (SRBD) is obstructive sleep apnea syndrome (OSAS). OSAS has been linked to hypertension, as well as an increased risk of arrhythmia, heart failure, coronary artery disease, stroke, and even mortality [4,11,12,13,14]. Cross-sectional studies in patients with coronary artery disease, heart failure, end-stage renal disease, and the general elderly population have found an association between LA size and OSAS [15,16,17,18]. OSAS is characterized by recurrent episodes of pharyngeal obstruction, either partial or complete, and produces negative intrathoracic pressure, which may lead to changes in the volume and stretching of the atria [4,19]. Additionally, OSAS results in intermittent hypoxemia and hypercapnia [4]. Intermittent hypoxia and post-apnea reoxygenation increase the myocardium’s oxidative stress, resulting in myocardial inflammation and remodeling [4,20]. Nocturnal hypoxemia episodes are one of the main reasons for developing complications associated with OSAS and are associated with an increase in mortality in patients with stable heart failure [21,22,23,24]. Additionally, the intermittent hypoxia and inflammation that occur in patients with OSAS activate hypoxia-inducible factors (HIF)-1, which may be associated with LA remodeling [25]. However, the detailed mechanism has not been thoroughly established until today. Because the apnea–hypopnea index (AHI) does not account for apnea depth and time, the oxygen desaturation index (ODI) and other desaturation severity measures may be better predictive of morbidity and death in individuals with OSAS than the AHI [22]. Given the importance of hypoxemic events in patients with OSAS, we set out to investigate the relationship between the ODI, the AHI, and other desaturation severity indices and LA remodeling in OSAS patients, as well as the possible mechanisms underlying hypoxemia and reoxygenation and their impact on LA remodeling.

Exosomes are released by a variety of cell types and are found in nearly all bodily fluids [26,27,28]. Increased interest in them has been sparked by the discovery of their role in intercellular genetic communication. HL-1 cells are contractile and exhibit phenotypic characteristics similar to those of adult atrial cardiomyocytes [29]. Our previous study demonstrated that exosomes isolated from the plasma of OSAS patients with AF influenced GJA1 gene expression in HL-1 cells [30]. The purpose of this study was to determine the effect of exosomes from patients with and without significant OSAS on LA remodeling by analyzing the expression of genes in HL-1 cells treated with exosomes. In OSAS patients, we hypothesized that intermittent hypoxia/reoxygenation (IHR) stimulation would increase reactive oxygen species (ROS) production and inflammatory cytokine release, resulting in LA apoptosis and remodeling. The purpose of this study was to determine the effect of IHR on the expression of HIF-1α and inflammation-associated cytokines in HL-1 cells and LA remodeling in OSAS patients, as well as the effect of exosomes from patients with and without significant OSAS on the expression of HIF-1α and inflammation-associated cytokines in order to better understand their relationship to LA remodeling.

## 2. Materials and Methods

### 2.1. Patient Enrollment and Sample Management

From May 2019 through to October 2021, patients with SRBD were enrolled in this study. Patients who had an acute infection, autoimmune disease, or malignancy or who were under the age of 30 were excluded from the study. Following enrollment in the study, participants underwent polysomnography (PSG) and peripheral blood (PB) sampling. Plasma from the PB was used to quantify and purify exosomes. The patients’ clinical features were analyzed, including age, sex, comorbidities, PSG, and LA size as assessed by echocardiography. Written informed consent was obtained from the participants before starting the study. This study was approved by the Institutional Review Board of Chang Gung Memorial Hospital (IRB number: 201801943B0, 7 January 2019) and conformed to the guidelines set forth by the Declaration of Helsinki.

### 2.2. Overnight PSG Study and Definition of SRBD Metrics

An overnight PSG study was performed on all individuals who were enrolled in this trial. The PSG procedures were described in detail in our previous research [30,31]. To summarize, the overnight PSG study was conducted in the sleep center of our hospital utilizing a commercial suite that has been standardized (Sandman Elite, Mallinckrodt Inc., St. Louis, MO, USA). Experienced technicians used defined criteria to record, analyze, and identify sleep parameters [32]. The absence of nasal airflow for at least 10 s was defined as apnea. Hypopnea was defined as a drop in nasal airflow of 30% or more for at least 10 s, accompanied by a decline in arterial SpO_2_ of more than 4% of the baseline level or arousal. Significant OSAS was defined as an ODI of ≥10 per hour associated with daytime drowsiness and sleep apneas. The ODI was calculated as the average number of desaturation occurrences each sleeping hour. Desaturation episodes were defined as a drop in O_2_ saturation of 4% or more in comparison with the pre-event baseline value lasting for at least 10 s.

### 2.3. Definition of LA Size by Echocardiography Measurement

In accordance with our previous echocardiography protocol, we used a Sonos 7500 (Live 3D Echo; Philips Medical Systems, Amsterdam, The Netherlands) to measure the size of the LA. This was carried out with conventional M-mode echocardiography that was performed perpendicular to the LA posterior wall’s long axis from the inner edge to inner edge, at the level of the aortic sinuses [33,34].

### 2.4. HL-1 Cell Culture, IHR Stimulation, and Measurement of Cell Viability, LDH Cytotoxicity, Intracellular ROS, and Cell Apoptosis

#### 2.4.1. HL-1 Cell Culture

Pooled primary cells of the HL-1 Cardiac Muscle Cell Line were purchased from SIGMA (SIGMA-ALDRICH Corp., St. Louis, MO, USA) and cultured in a mixture of Claycomb Basal Medium as previously described [35].

#### 2.4.2. In Vitro IHR Stimulation

HL-1 were seeded in 6-well plates (4 × 10^5^ cells/well) and then exposed to either normoxia or intermittent hypoxia/reoxygenation (IHR) stimulation. The custom-designed incubation chambers were connected to an external computer-controlled O_2_-CO_2_-N_2_ system. The detailed settings were described previously [36].

#### 2.4.3. Measurement of Cell Viability by a WST-1 Assay

For the last 2 h, WST-1 reagent was added as directed in the instruction manual (Roche, Mannheim, Germany). The viable cell mass was determined using an optical density measurement at 450 nm, with 600 nm serving as a reference wavelength.

#### 2.4.4. Measurement of Cytotoxicity by LDH Assay

The Pierce™ LDH Cytotoxicity Assay Kit was used to determine the cytotoxicity of the cells by measuring the amount of lactate dehydrogenase (LDH) released from the cytoplasm (Thermo Scientific, Waltham, MA, USA). The absorbance at 490 and 680 nm was determined using an ELISA reader.

#### 2.4.5. Measurement of Intracellular ROS

We used the ROS-sensitive probe 2′,7′-dichlorodihydrofluorescein diacetate to determine the intracellular ROS levels (H2DCFDA, SIGMA-ALDRICH Corp., St. Louis, MO USA). Following intermittent hypoxia/reoxygenation (IHR) stimulation, cells were incubated in the dark for 30 min with 5 M H2DCFDA and immediately analyzed using a flow cytometer set to 488 and 535 nm excitation and emission wavelengths, respectively (Beckman Cytomics™ FC500, Beckman Coulter, Brea, CA, USA).

#### 2.4.6. Flow Cytometry Analysis for Cell Apoptosis Measurement

The apoptosis rates of HL-1 cells were determined using flow cytometry following IHR stimulation using the CF™ 488A Annexin V/Propidium iodide (PI) apoptosis detection kit (Biotium, Fremont, CA, USA). The percentage of Annexin V and propidium iodide double-positive cells (late apoptosis) was determined in the FL1 channel using the Beckman Cytomics™ FC500 at excitation and emission wavelengths of 488 and 530 nm, respectively.

### 2.5. Exosome Isolation, Purification, Quantification, and Incubation in HL-1 Cells

The isolation, purification, and quantification of exosomes were carried out as previously described [37,38,39]. PB samples were collected from 95 patients with SRBD. The HL-1 cells were then treated for 24 h at 37 °C with 10 ug/mL exosomes obtained from patients with and without significant OSAS.

### 2.6. Real-Time Quantitative Reverse Transcriptase–Polymerase Chain Reaction Analysis (qRT-PCR) of mRNA Expression in HL-1 Cells

As previously described, HL-1 cells were isolated, RNA was extracted, cDNA was synthesized, and qRT-PCR was performed [40,41]. Supplementary Table S1 contains the sequences of the PCR primers. To obtain the relative threshold cycle (ΔCt), the expression levels of HIF-1α and inflammation-associated cytokines were normalized to the internal control GAPDH. Each reaction was carried out in duplicate.

### 2.7. Statistical Analysis

For all patients and patient subgroups, descriptive summaries are provided. Quantitative data are expressed as percentages, means ± standard deviation, or medians (interquartile range). The chi-square or Fisher’s exact test was used to compare categorical variables between patients with and without significant OSAS. Student’s *t*-test or the Mann–Whitney U-test were used to compare differences in continuous data. The 2^−^^ΔΔCt^ calculation was used to determine the fold changes in mRNA gene expression in HL-1 cells. Pearson’s correlation was used to examine the relationship between LA size in SRBD patients, mRNA gene expression in HL-1 cells, and PSG metrics in SRBD patients. We conducted all statistical analyses using SPSS version 17.0 software (SPSS, Chicago, IL, USA).

## 3. Results

### 3.1. Baseline Characteristics of Patients with and without Significant OSAS

Between May 2019 and October 2021, 154 patients with SRBD were prospectively enrolled. One hundred and nine (70.8%) had significant OSAS (ODI ≥ 10 per hour), and 45 (29.2%) did not. The differences between patients with and without significant OSAS are summarized in Table 1. Briefly, the patients with significant OSAS had a higher body mass index, AHI, and ODI; a lower lowest oxygen saturation (SpO_2_) and mean SpO_2_; and a larger LA size (all *p* < 0.05). Patients with and without significant OSAS had the same baseline characteristics, including age, sex, smoking habit, waistline, diabetes mellitus, hypertension, heart failure, stroke, coronary artery disease, thyroid disorder, and Epworth sleepiness scale.

### 3.2. Correlation between Polysomnography (PSG) Metrics and LA Size in Patients with SRBD

**Figure 1:**
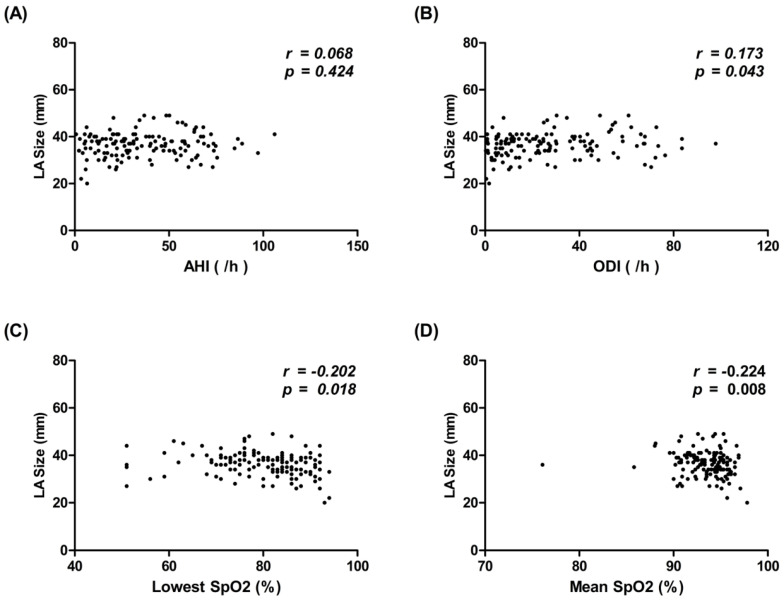
Correlation between LA size and the (**A**) AHI, (**B**) ODI, (**C**) lowest SpO_2_, and (**D**) mean SpO_2_ in patients with SRBD. The correlation was assessed by bivariate correlation analysis. The *r* and *p*-values indicate the correlation between LA size (expressed in units of mm) and AHI (expressed in units per hour), ODI (expressed in units per hour), lowest SpO_2_ (expressed in %), and mean SpO_2_ (expressed in). AHI, apnea–hypopnea index; LA, left atrial; ODI, oxygen desaturation index; SpO_2_, oxygen saturation; SRBD, sleep-related breathing disorder.

To evaluate the importance of hypoxemic events associated with LA remodeling in patients with SRBD, we analyzed the association between different PSG metrics and LA size. We found there was a positive correlation between LA size and ODI (*r* = 0.173, *p* = 0.043) and a negative correlation between LA size and the lowest SpO_2_ (*r* = −0.202, *p* = 0.018) and mean SpO_2_ (*r* = −0.224, *p* = 0.008) (Figure 1). There was no significant correlation between LA size and AHI.

### 3.3. Cell Viability, Lactate Dehydrogenase (LDH) Activity, ROS Levels, Apoptosis, and the Expression of HIF-1α and Inflammatory Cytokines of HL-1 Cells Exposed to IHR Stimulation or Normoxia

**Figure 2:**
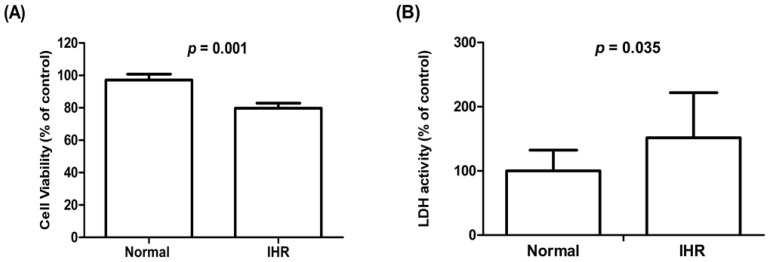
Cell viability (**A**) and LDH activity (**B**) in HL-1 cells exposed to IHR stimulation or normoxia on Day 3. The *y*-axis represents cell viability (% of control) in (**A**) and LDH activity (% of control) in (**B**). IHR, intermittent hypoxia/reoxygenation; LDH, lactate dehydrogenase.

**Figure 3:**
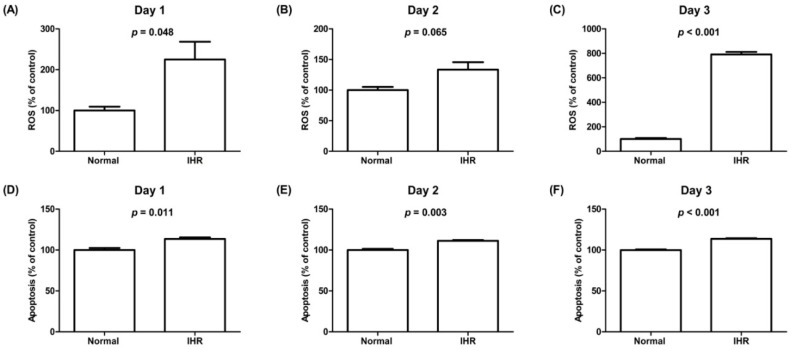
ROS activity (**A**–**C**) and apoptosis rates (**D**–**F**) in HL-1 cells exposed to IHR stimulation or normoxia on Days 1–3. The *y*-axis represents ROS levels (% of control) in (**A**–**C**) and apoptosis rates (% of control) in (**D**–**F**). IHR, intermittent hypoxia/reoxygenation; ROS, reactive oxygen species.

**Figure 4:**
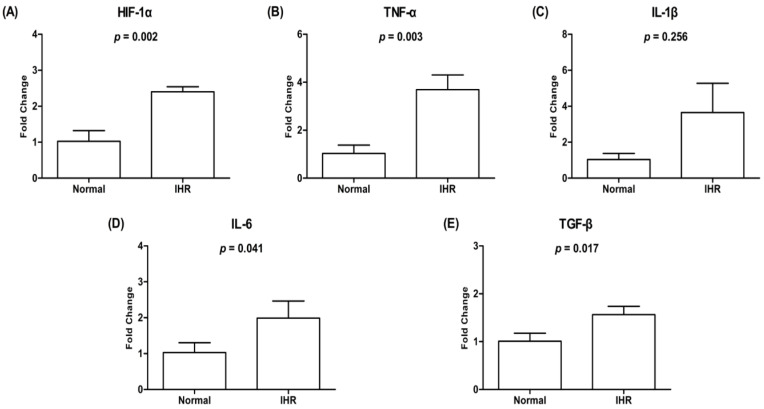
mRNA expression of HIF-1α (**A**) and inflammatory cytokines (**B**–**E**) of HL-1 cells exposed to IHR or normoxia. The *y*-axis represents the fold change in the mRNA expression level in HL-1 cells exposed to IHR stimulation compared with normoxia. HIF, hypoxia-inducible factor; IHR, intermittent hypoxia/reoxygenation; IL, interleukin; TGF, transforming growth factor; TNF, tumor necrosis factor.

To evaluate the impact of IHR on the morphologic and functional changes, we exposed HL-1 cells to IHR stimulation or normoxia, which showed decreased cell viability and increased LDH activity on Day 3, increased ROS levels on Day 1 and Day 3, and apoptosis from Day 1 to Day 3 in HL-1 cells exposed to IHR compared with normoxia (Figure 2 and Figure 3). The expression levels of HIF-1α, TNF-α, IL-6, and TGF-β were significantly higher in HL-1 cells exposed to IHR stimulation rather than normoxia (all *p* < 0.05) (Figure 4).

### 3.4. mRNA Gene Expression of HIF-1α and Inflammatory Cytokines in HL-1 Cells Treated with Exosomes Derived from Patients with and without Significant OSAS

**Figure 5:**
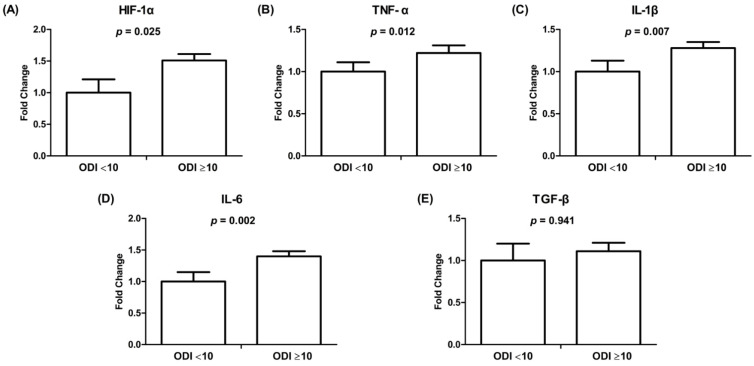
mRNA gene expression of HIF-1α (**A**) and inflammatory cytokines (**B**–**E**) in HL-1 cells treated with exosomes derived from patients with and without significant OSAS. The *y*-axis represents the fold change on mRNA expression levels in HL-1 cells incubated with exosomes derived from patients with significant OSAS (*n* = 74) compared with those without OSAS (*n* = 21). HIF, hypoxia-inducible factors; IL, interleukin; OSAS, obstructive sleep apnea syndrome; TGF, transforming growth factor; TNF, tumor necrosis factor.

To evaluate the effect and mechanism of intermittent hypoxemic events on atrial cardiomyocytes, HL-1 cells were treated with exosomes derived from 95 patients with and without significant OSAS. Patients with significant OSAS had higher mRNA expression levels of HIF-1α, TNF-α, IL-1β, and IL-6 (both *p* < 0.05) than patients without significant OSAS (Figure 5). There was no difference in the mRNA expression of TGF-β between patients with and without significant OSAS.

### 3.5. Correlation between mRNA Gene Expression of HIF-1α and Inflammatory Cytokines in HL-1 Cells Treated with Exosomes from Patients with SRDB, and PSG Metrics in Patients with SRDB

**Figure 6:**
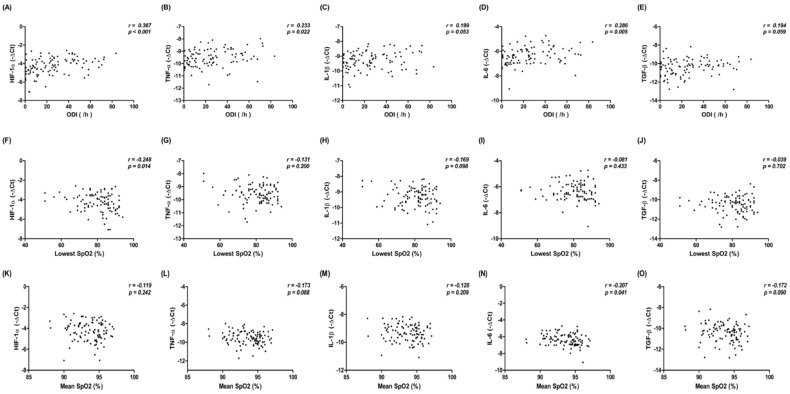
Correlation between the mRNA gene expression of HIF-1α and inflammatory cytokines in HL-1 cells treated with exosomes from 95 patients with SRDB, and PSG metrics, including ODI (**A**–**E**), lowest SpO_2_ (**F**–**J**), and mean SpO_2_ (**K**–**O**) in patients with SRDB. PSG, polysomnography; ODI, oxygen desaturation index; SpO_2_, oxygen desaturation; SRDB, sleep-related breathing disorder.

To investigate the possible impact of the severity of intermittent hypoxemia in 95 patients with SRDB on the expression of HIF-1a and inflammatory cytokines in HL-1 cells, we analyzed the correlation between PSG metrics in patients with SRDB and the expression of HIF-1a and inflammatory cytokines in HL-1 cells. We found there were significantly positive correlations between ODI and the expression of HIF-1α (*r* = 0.367, *p* < 0.001), TNF-α (*r* = 0.233, *p* = 0.022), and IL-6 (*r* = 0.286, *p* = 0.005); a significantly negative correlation between the lowest SpO_2_ and HIF-1a (*r* = −0.248, *p* = 0.014); and a significantly negative correlation between mean SpO_2_ and IL-6 (*r* = −0.207, *p* = 0.041). There was no significant correlation between ODI and the expression of IL-1β and TGF-β; between the lowest SpO_2_ and the expression of TNF-α, IL-1β, IL-6, and TGF-β; and between mean SpO_2_ and the expression of HIF-1α, TNF-α, IL-1β, and TGF-β (all *p* > 0.05) (Figure 6).

## 4. Discussion

There were several significant findings in this study. First, patients with significant OSAS had a larger LA size, and larger LA size was significantly associated with higher ODI, and lower lowest SpO_2_ and mean SpO_2_, but was not associated with AHI. Second, there were increases in the amount of LDH activity, ROS levels, apoptosis, and the expression of HIF-1α, TNF-α, IL-6, and TGF-β, and decreased cellular viability in HL-1 cells exposed to IHR stimulation compared with normoxia. Third, the expression of HIF-1α, TNF-α, IL-1β, and IL-6 was significantly increased in HL-1 cells treated with exosomes from patients with significant OSAS compared with those without significant OSAS. There was a significantly positive correlation between higher ODI and the increased expression of HIF-1α, TNF-α, and IL-6; a significantly negative correlation between a lower lowest SpO_2_ and increased expression of HIF-1α; and a significantly negative correlation between lower mean SpO_2_ and increased expression of IL-6.

A previous study showed that LA size was associated with the severity of OSAS and that AHI ≥ 15 was a significant predictor of LA enlargement [18]. In this study, ODI, mean SpO_2_, and lowest SpO_2_ but not AHI were significantly correlated with LA size. Respiratory arrest during sleep causes CO_2_ retention and hypoxemia, and desaturation episodes are one of the leading causes of the development of complications associated with OSAS [21,22,23]. Previous studies showed that ODI or desaturation severity was a better predictor than AHI for evaluating the clinical symptoms and risk of cerebrovascular and cardiovascular morbidities and mortality rates in patients with OSAS [22,42,43,44] The most critical limitations regarding AHI are that the morphological characteristics of abnormal respiratory events (i.e., duration and depth) are not taken into consideration. The physiological stress levels of different patients with similar AHI levels can be very different [22]. While the duration and depth of the apnea episodes increase, the frequency of AHI may decrease. Therefore, ODI and desaturation severity (i.e., lowest SpO_2_ and mean SpO_2_), which could be determined using a nocturnal pulse oximeter, maybe an earlier and better way to evaluate the pathophysiologic changes in patients with OSAS. Our study discovered that LA size was significantly associated with ODI but not with AHI in patients with SRBD, indicating that intermittent hypoxia plays a critical role in LA remodeling in these patients. Our findings suggest that in the study of LA remodeling, ODI may be a more useful and significant parameter for identifying high-risk patients with SRBD.

HIF-1 is activated under hypoxic conditions in various heart diseases, such as ischemic heart disease or heart failure, according to several studies [45,46,47]. In patients with OSAS, serum HIF-1α was found to be upregulated in recent studies [25,48,49]. HIF-1α expression was also linked to atrial fibrosis in rats induced with isoproterenol in a previous study [10]. Intermittent hypoxia can cause non-lethal ROS levels, which activate the HIF-1α promoter and drive HIF-1α transcription and function [50,51]. HIF is the master regulator of the hypoxia response and coordinates a transcriptional program that ensures optimal cellular adaptation to oxygen deprivation [52,53]. This study showed that IHR caused apoptosis and cellular damage in HL-1 cells with increased ROS levels, and the mRNA expression of HIF-1α. In addition, cell viability, LDH activity, and HIF-1α increased in HL-1 cells treated with exosomes from patients with significant OSAS compared with patients without OSAS. Moreover, the expression of HIF-1α was significantly and positively correlated with higher ODI and negatively correlated with the lowest SpO_2_ of patients with SRBD. Our study and previous studies revealed that intermittent hypoxemia-induced activation of HIF-1α signaling might play an essential role in the LA remodeling in patients with OSAS. However, it is unknown whether HIF-1α activation plays a critical role in myocardial protection against chronic hypoxemia to provide tissue tolerance against hypoxic injury. Further studies may investigate if targeting HIF-1α therapy could prevent LA remodeling in OSAS patients.

IHR activates pro-inflammatory cytokines including TNF-α and IL-1β, which have been demonstrated to promote HIF-1α accumulation and transcriptional activity [54]. In addition, through a signal transduction and activator of transcription (STAT3)-dependent mechanism, IL-6 and TGF increase the mRNA expression of HIF-1α [55,56]. TGF-β also enhances HIF-1α protein stability by decreasing PHD2 expression via Smads [57]. Activation of HIF-1α in mast cells, on the other hand, promoted the expression of IL-6 and TNF-α [58], but reduced HIF-1α expression, causing IL-1β expression to be hampered [59]. To summarize, hypoxia-induced HIF signaling and inflammation are mediated by the immune system’s complicated activities [53]. This study confirmed previous studies showing that IHR increases inflammatory cytokine expression. In individuals with SRBD, the production of inflammatory cytokines was linked to the severity of hypoxemia. More research is needed to determine the influence and interaction between HIF-1 and inflammation on LA remodeling in OSAS patients.

This study had some limitations. First, the sample size was insufficient to assess HIF-1’s predictive usefulness in LA remodeling. To corroborate the finding, larger cohort studies concentrating on verifying the role of HIF-1α expression in predicting LA remodeling should be conducted. Second, we did not look into the impact of therapy or other risk reduction strategies on HIF-1α, the expression of other inflammatory cytokines, and LA remodeling in OSAS patients. More research is needed to determine the impact of OSAS treatment on these biomarkers and clinical outcomes. Third, the left atrial volume index may be a more reliable method for determining the size of the left atrium than the LA dimension measured with conventional M-mode echocardiography. Regrettably, we were unable to provide data on LAVI in this study because it is not routinely measured in our daily practice. However, previous research and our previous study both indicated that there was a strong correlation between the LA’s dimension and left atrial volume index (r = 0.860, *p* < 0.001 in our previous study) [60,61]. Finally, we defined an ODI of >10 as moderate to severe OSAS according to a previous publication which demonstrated a sensitivity of 93% [62]. Although the results were statistically significant, underpowering may have occurred in the group with ODI < 10 due to the smaller number of cases (*n* = 45).

## 5. Conclusions

In conclusion, our study showed that intermittent hypoxemia plays an important role in LA remodeling in patients with SRBD. The ODI and other hypoxemia indices were significantly associated with LA remodeling. IHR stimulation and exosomes from patients with significant OSAS caused increased apoptosis, cellular damage, and ROS levels and decreased cellular viability in HL-1 cells. IHR stimulation and exosomes from significant OSAS patients also activated HIF-1α signaling and inflammatory cytokines, which were also significantly associated with ODI and other hypoxemia indices in patients with SRBD.

## Figures and Tables

**Table 1:** Baseline characteristics of obstructive sleep apnea syndrome patients with and without ODI ≥ 10.

Variables	ODI ≥ 10 (per hour) (*n* = 109)	ODI < 10 (per hour) (*n* = 45)	*p*-Value
Age	55.8 ± 11.0	53.3 ± 9.8	0.188
Sex (male)	81 (74.3%)	30 (66.7%)	0.336
Smoking	20 (18.3%)	11 (24.4%)	0.391
BMI	26.5 ± 3.3	24.2 ± 3.1	<0.001
Waistline	85.6 ± 8.3	81.7 ± 8.2	0.063
DM	13 (11.9%)	3 (6.7%)	0.331
HTN	42 (38.5%)	15 (33.3%)	0.543
HF	3 (2.8%)	0 (0%)	0.261
Stroke	4 (3.7%)	2 (4.4%)	0.821
CAD	5 (4.6%)	2 (4.4%)	0.969
AF	20 (18.3%)	6 (13.3)	0.518
Paroxysmal AF	9 (8.3%)	4 (8.9%)	
Persistent AF	11 (10.1%)	2 (4.4%)	
Thyroid disorder	2 (1.8%)	0 (0%)	0.360
Polysomnography			
Epworth sleepiness scale	9.3 (6.0–12.0)	8.0 (5.0–12.0)	0.254
AHI (per hour)	45.4 (27.7–43.2)	13.2 (5.7–20.2)	<0.001
ODI (per hour)	36.5 (19.7–48.4)	4.8 (2.7–7.1)	<0.001
Lowest SpO_2_ (%)	76.7 (72.0–84.0)	87.9 (86.0–91.0)	<0.001
Mean SpO_2_ (%)	92.8 (91.8–94.6)	94.7 (93.6–96.0)	<0.001
LA size by echocardiography	37.0 ± 5.0	34.6 ± 5.6	0.021

Data are expressed as the mean ± standard deviation, median (interquartile range), or number (%). AF, atrial fibrillation; AHI, apnea–hypopnea index; BMI, body mass index; CAD, coronary artery disease; DM, diabetes mellitus; HF, heart failure; HTN, hypertension; LA, left atrial; ODI, oxygen desaturation index; SpO_2_, oxygen saturation.

## Data Availability

The data underlying this article will be shared on reasonable request to the corresponding author.

## Supplementary Materials

The following are available online at https://www.mdpi.com/article/10.3390/life12020148/s1. Table S1: Quantitative real-time polymerase chain reaction primers.
