# Supplementary material for: The Impact of Intermittent Hypoxemia on Left Atrial Remodeling in Patients with Obstructive Sleep Apnea Syndrome
Source: Life (Basel). 2022 Jan 20;12(2):148. doi: 10.3390/life12020148 (PMC8874769; doi:10.3390/life12020148)
Supplement: Supplementary file 1 [file life-12-00148-s001.zip › life-1523090-supplementary.pdf]

life

MDPI

Article

The Impact of Intermittent Hypoxemia on Left Atrial Remodeling in Patients with Obstructive Sleep Apnea Syndrome

Yung-Lung Chen [1,2,3], Yung-Che Chen [2,4], Hui-Ting Wang [2,5], Ya-Ting Chang [2,6], Yen-Nan Fang [1], Shukai Hsueh [1], Wen-Hao Liu [1], Pei-Ting Lin [1], Po-Yuan Hsu [4], Mao-Chang Su [4], Kuo-Tung Huang [4] and Meng-Chih Lin [2,4,*]

[1] Department of Internal Medicine, Division of Cardiology, Kaohsiung Chang Gung Memorial Hospital, Kaohsiung 833, Taiwan; feymanchen@gmail.com (Y.-L.C.); wideopen1216@ocm.tw (Y.-N.F.); pather@cgmh.org.tw (S.H.); wenhao@cgmh.org.tw (W.-H.L.); r40391132@gmail.com (P.-T.L.)
[2] School of Medicine, College of Medicine, Chang Gung University, Taoyuan 333, Taiwan; yungchechen@yahoo.com.tw (Y.-C.C.); gardinea1983@gmail.com (H.-T.W.); emily0606@cgmh.org.tw (Y.-T.C.)
[3] Graduate Institute of Clinical Medical Sciences, College of Medicine, Chang Gung University, Taoyuan 333, Taiwan
[4] Department of Internal Medicine, Division of Pulmonary & Critical Care Medicine, Kaohsiung Chang Gung Memorial Hospital, Kaohsiung 833, Taiwan; hsupowan@yahoo.com.tw (P.-Y.H.); maochangsu@yahoo.com.tw (M.-C.S.); jelly@cgmh.org.tw (K.-T.H.)
[5] Emergency Department, Kaohsiung Chang Gung Memorial Hospital, Kaohsiung 833, Taiwan
[6] Department of Neurology, Kaohsiung Chang Gung Memorial Hospital, Kaohsiung 833, Taiwan
* Correspondence: mengchih@cloud.cgmh.org.tw; Tel.: +886-7-731-7123 (ext. 8300)

Citation: Chen, Y.-L.; Chen, Y.-C.; Wang, H.-T.; Chang, Y.-T.; Fang, Y.-N.; Hsueh, S.; Liu, W.-H.; Lin, P.-T.; Hsu, P.-Y.; Su, M.-C.; et al. The Impact of Intermittent Hypoxemia on Left Atrial Remodeling in Patients with Obstructive Sleep Apnea Syndrome. *Life* **2022**, *12*, 148. https://doi.org/10.3390/life12020148

Academic Editors: Emilio Nardi and Fabrizio Montecucco

Received: 8 December 2021
Accepted: 19 January 2022
Published: 20 January 2022

Publisher's Note: MDPI stays neutral with regard to jurisdictional claims in published maps and institutional affiliations.

Copyright: © 2022 by the authors. Licensee MDPI, Basel, Switzerland. This article is an open access article distributed under the terms and conditions of the Creative Commons Attribution (CC BY) license (https://creativecommons.org/licenses/by/4.0/).

Supplementary materials

Table S1. Primers used for quantitative real-time polymerase chain reaction.

Gene	Primer
HIF-1α F	5'- ACC TTC ATC GGA AAC TCC AAA G -3'
HIF-1α R	5'- ACT GTT AGG CTC AGG TGA ACT -3'
TNF-α F	5'- CCC TCA CAC TCA GAT CAT CTT CT -3'
TNF-α R	5'- GCT ACG ACG TGG GCT ACA G -3'
IL-1β F	5'- GCA ACT GTT CCT GAA CTC AAC T -3'
IL-1β R	5'- ATC TTT TGG GGT CCG TCA ACT -3'
IL-6 F	5'- TAG TCC TTC CTA CCC CAA TTT CC -3'
IL-6 R	5'- TTG GTC CTT AGC CAC TCC TTC -3'
TGF-β F	5'- CTC CCG TGG CTT CTA GTG C -3'
TGF-β R	5'- GCC TTA GTT TGG ACA GGA TCT G -3'
GAPDH F	5'- AGG TCG GTG TGA ACG GAT TTG -3 '
GAPDH R	5'- TGT AGA CCA TGT AGT TGA GGT CA -3 '
